# Evaluating the effect of crocin on contrast-induced nephropathy in rats 

**DOI:** 10.22038/AJP.2024.24786

**Published:** 2025

**Authors:** Mahnaz Zolfaghari Farajerdi, Fatemeh Rajabian, Bibi Marjan Razavi, Mahboobehr Ghasemzadeh Rahbarda, Abolfazl Khajavi Rad, Sakineh Amoueian, Hossein Hosseinzadeh

**Affiliations:** 1 *Department of Pharmacodynamics and Toxicology, School of Pharmacy, Mashhad University of Medical Sciences, Mashhad, Iran*; 2 *2Pharmaceutical Research Center, Pharmaceutical Technology Institute, Mashhad University of Medical Sciences, Mashhad, Iran*; 3 *Targeted Drug Delivery Research Center, Pharmaceutical Technology Institute, Mashhad University of Medical Sciences, Mashhad, Iran*; 4 *Department of Physiology, Faculty of Medicine, Mashhad University of Medical Sciences, Mashhad, Iran*; 5 *Neurogenic Inflammation Research Center, Mashhad University of Medical Sciences, Mashhad, Iran*; 6 *Department of Pathology, Emam Reza Hospital, Mashhad University of Medical Sciences, Mashhad, Iran*

**Keywords:** Antioxidants, Diatrizoate, Nephropathy, Indomethacin, L- NAME, Contrast medium

## Abstract

**Objective:**

Contrast-induced nephropathy (CIN) raises the risk of renal injury, but crocin, a saffron component, may improve kidney function. This study investigated crocin's protective effects against CIN in rats.

**Materials and Methods:**

Male Wistar rats were divided into eight groups: Sham, Control, Contrast medium (diatrizoate), Diatrizoate combined with crocin at 10, 20, or 40 mg/kg/day, Diatrizoate combined with N-acetylcysteine (NAC) at 125 mg/kg/day, and Crocin alone at 40 mg/kg/day. Water deprivation began on day 5 for 48 hr, except for the sham and crocin alone groups. Indomethacin and N(ω)-nitro-L-arginine methyl ester were administered after 40 hr of dehydration. Rats were sacrificed on the eighth day, and blood and kidney samples were collected.

**Results:**

Diatrizoate increased serum creatinine and blood urea nitrogen levels, elevated malondialdehyde levels, and reduced glutathione in renal tissue. Crocin reversed these effects. Diatrizoate caused severe tubular necrosis, proteinaceous casts, medullary congestion, and interstitial edema in kidney tissue. Crocin (20 and 40 mg/kg) significantly reduced tubular necrosis, and doses of 10 and 40 mg/kg reduced interstitial edema. NAC significantly improved histopathological damage, biochemical factors, and oxidative stress. The crocin alone group showed no significant changes.

**Conclusion:**

Diatrizoate induces nephrotoxicity by enhancing oxidative stress in rats, and crocin has a protective effect against it. Crocin mitigates both tissue and biochemical damage inflicted by diatrizoate.

## Introduction

Acute kidney injury known as contrast-induced nephropathy, is caused by the intra-arterial or intravenous use of contrast media (James et al., 2013). Although mostly reversible, it might be accompanying adverse effects. With the gradual rise in the usage of contrast media, it is encountered as a more frequent clinical issue in practice. Hydrating the patient is the best-known prevention strategy against the emergence of contrast-induced nephropathy (Topaloğlu et al., 2019). The pathophysiology of contrast-induced nephropathy has not been sufficiently recognized. The factors considered to be effective in pathogenesis include alterations in renal microcirculation, endothelial dysfunction, renal medullar hypoxia caused by reduced blood flow due to vasoconstriction of the renal arteries, direct tubular toxicity caused by contrast media via oxidative stress, inflammation, autophagy, and apoptosis (Rajabian et al., 2023; Topaloğlu et al., 2019).

The previous documents reported that contrast-induced nephropathy or renal failure might be prevented with several herbal medicines or their main components including black seed, curcumin, garlic, ginger, silymarin, green tea, grape, pomegranate, thymoquinone, saffron, and resveratrol (Boozari and Hosseinzadeh, 2017, 2021).

*Crocus sativus* L. (saffron) is a perennial plant member of the Iridaceae family that is mostly grown in Iran (Alavizadeh and Hosseinzadeh, 2014). Saffron has long been used in traditional medicine as an aphrodisiac, antispasmodic, carminative, anticatarrhal, diaphoretic, eupeptic, expectorant, nerve sedative, stimulant, and stomachic agent, aside from being used as a food colorant and spice (Amin et al., 2015; Hosseinzadeh and Nassiri‐Asl, 2013). Three key saffron constituents -crocin (crocetin glycoside), safranal, and crocetin- are in charge of the saffron pharmacological effects (Rezaee and Hosseinzadeh, 2013). There are numerous biological effects attributed to saffron and its constituents, including antioxidant (Hosseinzadeh et al., 2009a; Hosseinzadeh and Sadeghnia, 2005; Hosseinzadeh et al., 2009b), analgesic (Amin and Hosseinzadeh, 2015), anti-inflammatory (Zeinali et al., 2019), antiasthmatic (Boskabady et al., 2010; Boskabady et al., 2011), antidote (Razavi and Hosseinzadeh, 2015), anxiolytics and antidepressants (Asrari et al., 2018; Hosseinzadeh et al., 2007; Rahbardar and Hosseinzadeh, 2021), antirheumatic (Nakisa and Rahbardar, 2021), memory and learning enhancing (Hosseinzadeh et al., 2012), antitussive (Razavi and Hosseinzadeh, 2015), reducing withdrawal syndrome (Nassiri-Asl and Hosseinzadeh, 2015; Shoja et al., 2018), anticancer (Boozari and Hosseinzadeh, 2022), and hypotensive (Razavi and Hosseinzadeh, 2017) properties. Saffron has also been found to increase the diffusion of oxygen in several tissues (Moshiri et al., 2015). The renoprotective effects of saffron or its main components have been presented in several studies. For instance, Rajabian et al. reported that pretreating HEK-293 cells with trans-sodium crocetinate has an antioxidant impact that reduces contrast-induced cytotoxicity and modifies the extracellular signal-regulated kinase (ERK), apoptosis, and autophagy pathways (Rajabian et al., 2023). Renal ischemia-reperfusion-induced oxidative injury in rats can be prevented by aqueous saffron extract and its active compound, crocin (Hosseinzadeh et al., 2005). Furthermore, the antilithiatic property of crocin has been shown in rats with ethylene glycol-induced lithiasis (Ghaeni et al., 2014). 

This research sought to investigate how crocin impacts contrast-induced nephropathy in rats by considering crocin's properties and the mechanisms underlying the condition's pathophysiology. The study was structured to assess how effectively crocin can prevent nephropathy development in a rat model of contrast-induced nephropathy.

## Materials and Methods

### Chemicals

N-acetylcysteine (NAC) was purchased from Tinab Shimi, Iran. Indomethacin and N(ω)-nitro-L-arginine methyl ester (L-NAME) were obtained from Sigma-Aldrich, USA. Crocin and 5, 50-dithiobis 2-nitrobenzoic acid (DTNB) were obtained from Sigma Company, Germany. Tween 20%, potassium chloride, thiobarbituric acid, and phosphoric acid were acquired from Merck, Germany. Meglumine/sodium diatrizoate (Urografin 76% w/v; density 1.5 mg/cm^3^) was obtained from Bayer, Turkey. Formaldehyde solution 10% and n-butanol were purchased from DRM CHEM, Iran. SL-urea assay kit and SL-creatinine assay kit were purchased from Mancompany, Iran.

### Animals

In this experimental study, 48 male Wistar rats of 230-250 g weight were prepared and kept in standard cages at a temperature of 23±2°C and in a 12 hr light/12 hr darkness cycle in the animal room of the School of Pharmacy, Mashhad Iran. During the storage period, the animals had free access to food and water. Three Wistar rats in the contrast medium group died during the experiment.

All animal experiments adhered to the guidelines set forth by the Mashhad Ethics Committee (No: 931316, dated 15.03.2017).

### Study protocol

**Figure 1:**
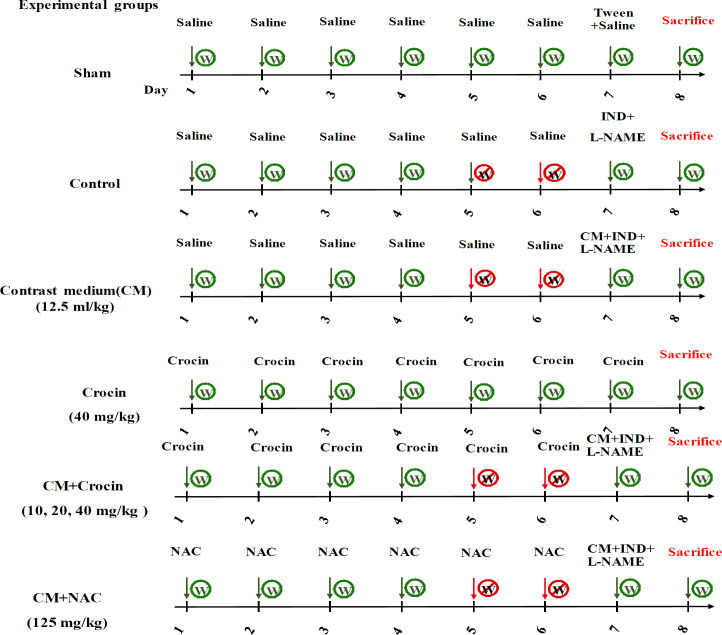
Schematic diagram of the study protocol. IND: indomethacin, L- NAME: N(ω)-nitro-L-arginine methyl ester, CM: contrast medium, and NAC: N-acetylcysteine.

1-Sham group: This group was defined to observe and determine the effects of vehicles, the animals in this group were not exposed to dehydration. The rats in this group received normal saline instead of crocin for one week, and on the seventh day of the experiment, they received normal saline plus 30 µL of Tween 20% (instead of indomethacin) and normal saline (instead of L-NAME and contrast medium) (Figure 1).

2-Control group: These rats received normal saline instead of crocin for 7 days. On the fifth day after the start of the study, they were dehydrated for 48 hr, and on the seventh day, they received indomethacin (10 mg/kg), L-NAME (10 mg/kg), and normal saline (instead of contrast medium).

3-Contrast medium group (diatrizoate): These rats were dehydrated for 48 hr (days 5-7). During the study period, these animals received normal saline instead of crocin (for 7 days). On the seventh day (after 40 hr dehydration), they were administered with indomethacin (10 mg/kg), L-NAME (10 mg/kg), and diatrizoate (12.5 mL/kg or 25 mg/kg according to its density) (Topaloğlu et al., 2019).

4-6-Crocin (10, 20, and 40 mg/kg/day) groups: These rats were exposed to dehydration for 48 hr (days 5-7) and treated with crocin for a week. On the seventh day of the study, indomethacin (10 mg/kg), L-NAME (10 mg/kg), and diatrizoate (12.5 mL/kg) were administered. Crocin doses were selected according to previous studies (Mohammadzadeh et al., 2022; Samarghandian et al., 2016).

7- NAC (125 mg/kg/day) group: This group was used as a positive control, and its administration protocol was identical to that of groups 4-6, except that they received NAC instead of crocin. The NAC dose was selected according to our pilot study.

8-In the Crocin alone group (40 mg/kg), the objective was to evaluate the isolated effects of crocin. Rats in this group received crocin throughout the study period (7 days) with unlimited access to water.

On the seventh day of the study, 8 hr following the administration of L-NAME, indomethacin, and diatrizoate, all rats from various groups were given access to water. On the eighth day of the experiment, the rats were euthanized, through decapitation using the guillotine and samples of blood and kidney tissue were collected for analysis (Topaloğlu et al., 2019). The right kidney samples were preserved by initially storing them in liquid nitrogen and subsequently transferring them to a freezer set at -80°C until analysis. For histological examinations, the left kidneys were immersed in a 10% formaldehyde solution for fixation.

It is important to mention that each group consisted of six rats, and all administrations were performed intraperitoneally (i.p.).

### Assessing kidney function

Kidney function was assessed by analyzing blood serum samples to measure the levels of blood urea nitrogen (BUN) using an SL-urea assay kit by the Enzymatic-UV-kinetic method, and creatinine levels using an SL-creatinine assay kit by the Jaffe-kinetic method (Mindray BS-800M) (Ephraim et al., 2020; Javadi et al., 2014). 

### Determination of lipid peroxidation

In an acidic environment, MDA (malondialdehyde) reacts with thiobarbituric acid to produce a pink-colored complex which can be quantified spectrophotometrically with a maximum absorption wavelength of 532 nm (Mihara and Uchiyama, 1978).

Initially, homogeneous tissue samples (10%) were prepared by blending them in 1.15% cold KCl using a homogenizer (Homogenizer POLYTRON® PT 10-35, Kinematica, Switzerland). Subsequently, 0.5 ml of this homogenate was combined with 3 ml of 1% phosphoric acid and 1 ml of 0.6% TBA (thiobarbituric acid) solution, and then subjected to boiling water for 45 min. After cooling the mixture, 4 ml of n-butanol was added and vortexed for 1 min to extract the colored complex (Vortex, VTX-3000, Scientfica, Japan). The solution was then centrifuged at 3000 g for 10 min (Centrifuge Minispin 5810R, Eppendorf, Germany). The organic phase of each sample was transferred into a new tube, and the absorbance was measured at a wavelength of 532 nm using a spectrophotometer (Jenway 6105 UV/Vis spectrophotometer, UK). A standard curve was generated within the concentration range of 0-100 nmol/ml of MDA. Ultimately, the quantity of MDA is expressed in terms of nmol per gram of tissue (Rahbardar et al., 2022).

### Determination of glutathione (GSH) level

The interaction of free sulfhydryl groups with DTNB in an alkaline media serves as the foundation for this test. The created colored complex has a maximum absorption at 412 nm (Moron et al., 1979).

Initially, a 10% homogenate of each tissue sample (100 mg) was prepared using phosphate buffer (pH 7.4). Subsequently, the homogenized samples were mixed with 10% tricarboxylic acid at a 1:1 ratio and then centrifuged at 2500 g for 10 min following vortexing (utilizing a Centrifuge Universal 320R, Heittich, Germany). The upper phase of each sample was then separated and combined with 2 ml of phosphate buffer (pH 8). To this mixture, 0.5 ml of 0.04% DTNB reagent was added, and the absorbance was measured at a wavelength of 412 nm using a spectrophotometer. Following the construction of a standard curve for GSH within the concentration range of 0-300 nmol/ml, the quantity of GSH was calculated and reported in terms of nmol per gram of tissue (Ghasemzadeh Rahbardar et al., 2020).

### Histopathology

The kidney tissues underwent embedding in paraffin, subsequent sectioning, and staining with hematoxylin and eosin. A proficient pathologist then examined the histopathological slides under a light microscope, evaluating differences among the groups on a scale ranging from 0 to 3. Here, a score of 0 represents normal tissue, while a score of 3 indicates the most severe injury.

### Statistical analysis

Statistical analysis was performed using Prism 8 software. Results are presented as Mean±standard deviation (SD). One-way ANOVA and Tukey-Kramer posttest were employed for comparing data between different groups. The Shapiro-Wilk test was used for assessment of the normality of the data. A significance level of p<0.05 was considered statistically significant for all groups. Additionally, the non-parametric Kruskal-Wallis test was used to analyze pathology data, and the results are reported as median (Interquartile Range). 

## Results

### Effect of crocin and diatrizoate on serum creatinine and BUN levels

**Figure 2:**
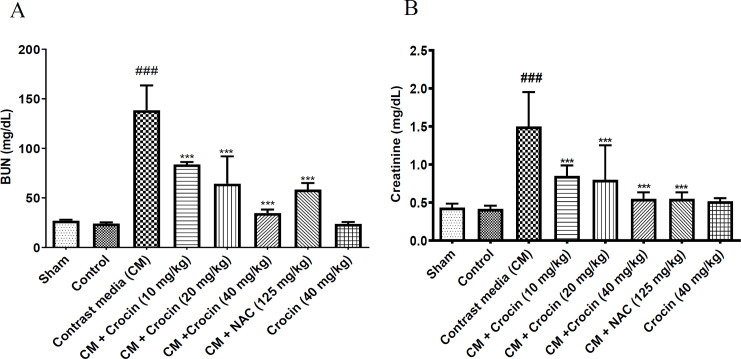
Effect of crocin and diatrizoate on A: BUN and B: serum creatinine levels. Contrast medium (12.5 mg/kg), crocin (10, 20, and 40 mg/kg), and NAC (125 mg/kg) were administered to rats. Data are Mean±SD (n=6). ANOVA test and Tukey-Kramer posttest were used to check the statistical difference. ###p<0.001 compared to sham group and ***p<0.001 compared to contrast medium group. CM: contrast medium, NAC: N-acetylcysteine.

As depicted in Figure 2, there were no discernible differences in serum creatinine and BUN levels between the sham and control groups. Following the administration of diatrizoate (12.5 ml/kg), a significant increase in serum BUN levels was observed compared to the sham group (p<0.001). Concurrent administration of crocin at all three doses (10, 20, and 40 mg/kg) alongside diatrizoate led to a notable reduction in BUN levels compared to the contrast medium group (p<0.001). Similarly, NAC at a dose of 125 mg/kg significantly mitigated the elevation of BUN compared to the contrast medium group (p<0.001). Notably, crocin at a dose of 40 mg/kg did not induce significant changes in BUN levels compared to the sham group (Figure 2A).

A single injection of diatrizoate (12.5 ml/kg) in combination with L-NAME and indomethacin resulted in a significant increase in serum creatinine levels compared to the sham group (p<0.001). Administration of crocin at all three doses (10, 20, and 40 mg/kg) concurrently with diatrizoate led to a significant decrease in serum creatinine levels compared to the contrast medium group (p<0.001). Furthermore, NAC at a dose of 125 mg/kg significantly attenuated the elevation of serum creatinine caused by diatrizoate injection (p<0.001). Interestingly, administration of crocin at a dose of 40 mg/kg in rats did not lead to significant changes in serum creatinine levels compared to the sham group (Figure 2B).

### Effect of crocin and diatrizoate on renal MDA and GSH levels

**Figure 3:**
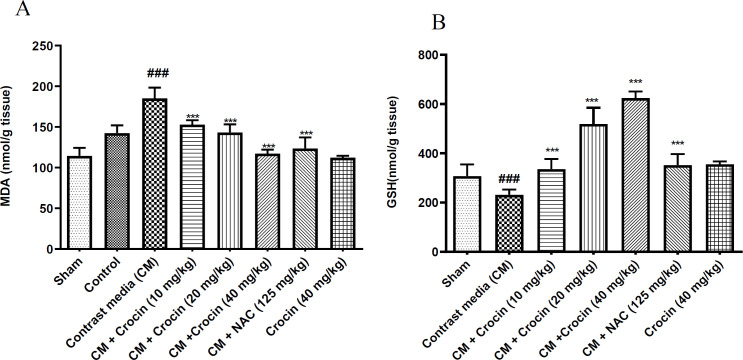
Effect of crocin and diatrizoate on renal A: MDA and B: GSH levels. Contrast medium (12.5 mg/kg), crocin (10, 20, and 40 mg/kg), and NAC (125 mg/kg) were administered to rats. Data are Mean±SD (n=6). ANOVA test and Tukey-Kramer posttest were used to check the statistical difference. ###p<0.001 compared to sham group and ***p<0.001 compared to contrast medium group. CM: contrast medium, NAC: N-acetylcysteine.

The levels of MDA and GSH in kidney tissue were found to be comparable between the control and sham groups. However, a single dose injection of diatrizoate (12.5 ml/kg) significantly elevated MDA levels in kidney tissues compared to the sham group (p<0.001). Administration of crocin at all three doses (10, 20, and 40 mg/kg), as well as NAC (125 mg/kg) resulted in a significant decrease in renal MDA content compared to the contrast medium group (p<0.001). Notably, there was no significant difference between the crocin 40 mg/kg alone group and the sham group (Figure 3A).

Similarly, injection of diatrizoate (12.5 ml/kg) led to a significant reduction in the GSH content of kidney tissue compared to the sham group (p<0.001). However, administration of crocin at all three doses (10, 20, and 40 mg/kg) and NAC (125 mg/kg) resulted in a significant increase in kidney GSH content compared to the contrast medium group (p<0.001). Once again, there were no significant differences between the group receiving crocin 40 mg/kg alone and the sham group regarding GSH levels (Figure 3B).

### Effect of crocin and diatrizoate on renal histology

**Figure 4:**
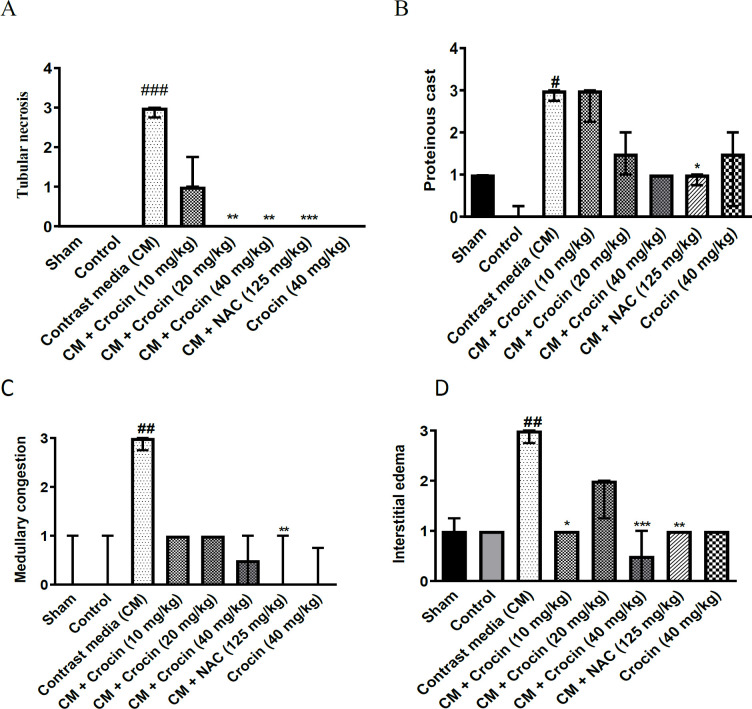
Effect of crocin and diatrizoate on renal histology: A: tubular necrosis B: proteinaceous casts, C: medullary congestion, and D: interstitial edema. Contrast medium (12.5 mg/kg), crocin (10, 20, and 40 mg/kg), and NAC (125 mg/kg) were administered to rats. Data are median ± IQR (n=6). ANOVA test and non-parametric Kruskal-Wallis test were used to check the statistical difference. ###p<0.001, ##p<0.01, and #p<0.05 compared to sham group and ***p<0.001, **p<0.01, and *p<0.05 compared to contrast medium group. CM: contrast medium, and NAC: N-acetylcysteine.

As illustrated in Figure 4, renal histology in the control, sham, and crocin 40 mg/kg alone groups exhibited no discernible differences. However, administration of diatrizoate (12.5 ml/kg) induced severe tubular necrosis compared to the sham group (p<0.001). Crocin at doses of 20 and 40 mg/kg significantly mitigated tubular necrosis (p<0.01). No statistically significant difference was observed at the crocin dose of 10 mg/kg. Additionally, administration of NAC (125 mg/kg) significantly reduced tubular necrosis compared to the contrast medium group (p<0.001) (Figure 4A).

**Figure 5:**
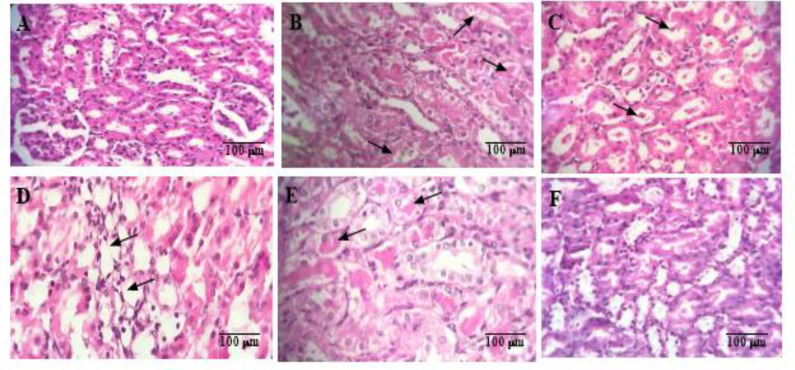
Samples of tissue pathology images of different groups stained with hematoxylin and eosin (40_X_ magnification). A: Section of the sham group without histopathological damage. B: Section of contrast medium group with tubular necrosis and proteinaceous casts. C: Section of contrast medium group with proteinaceous casts. D: Section of contrast medium group with interstitial edema. E: Section of contrast medium+crocin 10 mg/kg group with proteinaceous casts. F: Section of crocin 40 mg/kg alone group without histopathological damage.

Moreover, diatrizoate (12.5 ml/kg) induced the formation of proteinaceous casts in kidney tissue compared to the sham group (p<0.05). While the groups receiving crocin at all three doses showed a reduction in proteinaceous casts, it was not statistically significant compared to the sham. However, administration of NAC (125 mg/kg) led to a significant decrease in proteinaceous casts compared to the contrast medium group (p<0.05) (Figure 4B, 5).

Additionally, diatrizoate caused medullary congestion in kidney tissue compared to the sham group (p<0.01). Although there was a reduction in this complication in the groups receiving crocin at all three doses, it did not reach statistical significance compared to the contrast medium (CM). Administration of NAC (125 mg/kg) significantly decreased medullary congestion compared to the contrast medium group (p<0.01) (Figure 4C).

Furthermore, diatrizoate-induced interstitial edema in kidney tissue compared to the sham group (p<0.01). Crocin at doses of 10 mg/kg (p<0.05) and 40 mg/kg (p<0.001) significantly reduced this complication, while the dose of 20 mg/kg had no significant effect compared to contrast medium (CM). Administration of NAC at a dose of 125 mg/kg also led to a significant decrease in interstitial edema compared to the contrast medium group (p<0.01) (Figure 4D).

## Discussion

The current study examined the probable renoprotective effects of crocin against contrast-induced renal toxicity in rats. According to the results of this study, diatrizoate caused kidney damage in rats which was manifested as a sharp increase in serum creatinine and BUN levels. Furthermore, the data showed that diatrizoate caused a noticeable upturn in the amounts of MDA and a decline in the content of GSH in the kidney tissue, although crocin reversed these alterations. On the other hand, the results of the pathology test of rat kidney samples showed that diatrizoate caused severe tubular necrosis, formation of proteinaceous casts in the kidney tissue, medullary congestion, and interstitial edema in the kidney tissue. Crocin at doses of 20 and 40 mg/kg caused a significant decrease in tubular necrosis and at doses of 10 and 40 mg/kg triggered a decrease in interstitial edema in kidney tissue, but it had no significant effect on the formation of proteinaceous casts in kidney tissue or medullary congestion.

Kidney injury models are divided into acute and chronic kidney injury (Mohammed-Ali et al., 2017). Contrast media induces acute kidney injury which is one of the main problems faced by patients in medical imaging. In this model, indomethacin and L-NAME were used to prevent the synthesis of prostaglandins and nitric oxide, respectively, which revealed the vasoconstriction properties of the contrast media. Therefore, indomethacin and L-NAME block medullary vasodilator mechanisms and reduce medullary blood flow to approximately half of the baseline (Topaloğlu et al., 2019). Furthermore, the administration of contrast media in addition to indomethacin and L-NAME leads to extensive necrosis of the ascending tubule of the renal medulla which may be due to a further decrease in glomerular filtration rate (by leakage, obstruction, or tubuloglomerular reabsorption) or delayed recovery of renal function. All of these factors contribute to the increased damage caused by contrast media, which is a suitable model for acute kidney injury (Topaloğlu et al., 2019). 

In a study conducted by Khaleel et al., the administration of diatrizoate (6 ml/kg, i.p. + indomethacin + L-NAME) resulted in elevated levels of serum creatinine and BUN in rats. Additionally, it led to increased MDA levels and decreased GSH levels in kidney tissue. Histological examination revealed that diatrizoate increased the infiltration of inflammatory cells, interstitial bleeding, and glomerular hypercellularity (Khaleel et al., 2019). It has been noted that the injection of diatrizoate with L-NAME and indomethacin increased the serum concentrations of MDA, creatinine, and BUN. In addition, it resulted in medullary congestion, proteinaceous casts, and tubular necrosis in kidney tissue (Topaloğlu et al., 2019). In this study, diatrizoate increased serum creatinine and BUN levels, increased MDA, and decreased the amount of renal GSH in kidney tissue. Furthermore, pathological changes were observed, including tubular necrosis, proteinaceous casts, medullary congestion, and interstitial edema.

In contrast, a study conducted by Yarijani et al. found that administration of crocin (at a dose of 100 mg/kg, intraperitoneally for 7 days) resulted in a reduction of gentamicin-induced nephrotoxicity in rats. This administration of crocin led to decreased plasma levels of creatinine and BUN, along with reduced levels of MDA in kidney tissue. Furthermore, crocin administration moderately mitigated gentamicin-induced cellular damage which included glomerular atrophy, cellular desalination, tubular fibrosis and necrosis, epithelial edema of proximal tubules, vascular congestion, perivascular edema, and intratubular proteinaceous casts (Yarijani et al., 2016). In line with the previous study, our findings showed that crocin (10, 20, and 40 mg/kg) led to a decrease in serum creatinine and BUN, and renal MDA levels and an increase in GSH content. Moreover, in the pathology results of this study, crocin decreased tubular necrosis and interstitial edema in kidney tissue, and no change in the formation of proteinaceous casts which might be due to lower concentrations of crocin used in our study compared with the previous study. 

Furthermore, the effects of saffron extract (30 mg/kg, i.p.) and crocin (30 mg/kg, i.p.) on chronic stress (6 hr per day) generated by oxidative stress in the kidney were studied in rats for 21 days. Saffron extract and crocin decreased the level of MDA, and the activity of antioxidant enzymes, including glutathione reductase (GR), glutathione peroxidase (GPx), and superoxide dismutase (SOD) in stressed animals (Bandegi et al., 2014). In a study by Hosseinzadeh et al., the protective effect of crocin on oxidative damage caused by kidney ischemia/reperfusion in rats was investigated. Crocin pretreatment (50, 200, and 400 mg/kg, i.p.) decreased MDA levels and increased GSH concentration (Hosseinzadeh et al., 2005). In addition, crocin improved renal damage caused by carbon tetrachloride in rats by improving metabolic enzymes and reducing oxidative stress, inflammation, and apoptosis (Hassan et al., 2015).

Moreover, our team used NAC as a positive control. The administration of NAC along with diatrizoate considerably reduced serum creatinine and BUN levels, oxidative stress (decreased renal MDA levels and enhanced GSH amounts), and histopathological damage, including tubular necrosis, proteinaceous casts, medullary congestion, and interstitial edema.

It has also been reported that rapid intravenous hydration with sodium bicarbonate and NAC prevented contrast-induced nephropathy in patients undergoing emergency percutaneous coronary intervention (Recio-Mayoral et al., 2007). Another research reported that NAC treatment enhanced renal function in streptozotocin-induced diabetic nephropathy by reducing renal MDA levels and increasing GSH levels in rats (Mahajan et al., 2020). In the current investigation, NAC appears to be considerably more effective than crocin in improving some histological factors, including proteinaceous casts and medullary congestion in kidney tissue.

The findings suggest that kidney injury triggered by contrast media stems from oxidative stress, evidenced by increased MDA levels and decreased GSH content. Crocin mitigates both tissue and biochemical damage inflicted by contrast media, likely owing to its antioxidant characteristics.
